# Rapid loss of early antigen-presenting activity of lymph node dendritic cells against Ag85A protein following *Mycobacterium bovis* BCG infection

**DOI:** 10.1186/s12865-018-0258-8

**Published:** 2018-06-25

**Authors:** Zhengzhong Xu, Aihong Xia, Xin Li, Zhaocheng Zhu, Yechi Shen, Shanshan Jin, Tian Lan, Yuqing Xie, Han Wu, Chuang Meng, Lin Sun, Yuelan Yin, Xiang Chen, Xinan Jiao

**Affiliations:** 1 Jiangsu Key Laboratory of Zoonosis, Yangzhou University, No. 48 Wenhui East Road, Yangzhou, 225009 Jiangsu China; 2 Key Laboratory of Prevention and Control of Biological Hazard Factors (Animal Origin) for Agrifood Safety and Quality, MOA of China, Yangzhou University, Yangzhou, China; 3 Jiangsu Co-Innovation Center for Prevention and Control of Important Animal Infectious Diseases and Zoonoses, Yangzhou University, Yangzhou, China

**Keywords:** Ag-presenting activity, Dendritic cell, *M. bovis* BCG, Major histocompatibility complex class II, In vivo

## Abstract

**Background:**

Control of *Mycobacterium tuberculosis* (*Mtb*) infection requires CD4^+^ T-cell responses and major histocompatibility complex class II (MHC II) presentation of *Mtb* antigens (Ags). Dendritic cells (DCs) are the most potent of the Ag-presenting cells and are central to the initiation of T-cell immune responses. Much research has indicated that DCs play an important role in anti-mycobacterial immune responses at early infection time points, but the kinetics of Ag presentation by these cells during these events are incompletely understood.

**Results:**

In the present study, we evaluated in vivo dynamics of early Ag presentation by murine lymph-node (LN) DCs in response to *Mycobacterium bovis* bacillus Calmette–Guérin (BCG) Ag85A protein. Results showed that the early Ag-presenting activity of murine DCs induced by *M. bovis* BCG Ag85A protein in vivo was transient, appearing at 4 h and being barely detectable at 72 h. The transcription levels of CIITA, MHC II and the expression of MHC II molecule on the cell surface increased following BCG infection. Moreover, BCG was found to survive within the inguinal LN DC pool, representing a continuing source of mycobacterial Ag85A protein, with which LN DCs formed Ag85A peptide-MHCII complexes in vivo.

**Conclusions:**

Our results demonstrate that a decrease in Ag85A peptide production as a result of the inhibition of Ag processing to is largely responsible for the short duration of Ag presentation by LN DCs during BCG infection in vivo*.*

## Background

Tuberculosis (TB), caused by infection with *Mycobacterium tuberculosis* (*Mtb*), remains a major disease worldwide and is the leading infectious disease in terms of mortality, being responsible for an estimated 1.3 million deaths globally in 2016. Moreover, in the same year, there were an estimated 10.4 million new cases of active TB worldwide. *Mycobacterium bovis* bacillus Calmette–Guérin (BCG) is the only TB vaccine for humans in current use, but its efficacy is insufficient to prevent pulmonary TB in adults and reactivation of latent *Mtb* infection [1]. BCG vaccination mainly induces effector, rather than central, memory T cells, which are maintained for a shorter period, explaining the limited duration of protection afforded [2, 3].

CD4^+^ T-cell responses and the production of interferon gamma (IFN-γ) are particularly important to the containment of *Mtb* infection [4, 5]. Dendritic cells (DCs) represent the bridge between the innate and adaptive immune responses and specifically strengthen the cellular immune response against mycobacterial infections [6, 7]. Thus, the mechanisms involved in major histocompatibility complex class II (MHC II) antigen (Ag) processing and presentation, which are required for CD4^+^ T-cell activation, are crucial for controlling *Mtb* infection [8]. Much research has indicated that DCs play an important role in anti-mycobacterial immune responses in the early stages of infection, but little is known of the kinetics of Ag presentation by these cells soon after *M. bovis* BCG exposure. Indeed, efforts to understand the basis of protective immunity against *Mtb* have led us the examinntion of even earlier infection time points. We previously investigated the Ag-presenting cell (APC) functions of murine DCs during the first 2 weeks following intravenous administration of recombinant BCG (rBCG) expressing the *Escherichia coli* MalE protein as a reporter Ag [9]. However, this process has not yet been directly examined in lymph node (LN) DCs using an endogenous *M. bovis* BCG Ag.

In the present study, we evaluated the in vivo dynamics of early Ag presentation by murine inguinal LN DCs in response to *M. bovis* BCG. The results showed that the early Ag-presenting activity of murine DCs induced by *M. bovis* BCG Ag85A protein in vivo was transient and that the inhibition of Ag processing due to the decreased production of Ag85A peptide is the primary reason for the rapid loss of Ag85A peptide-MHC II complexes.

## Results

### Stimulation of Ag85A-specific IFN-γ production in BCG-infected mice

**Fig. 1:**
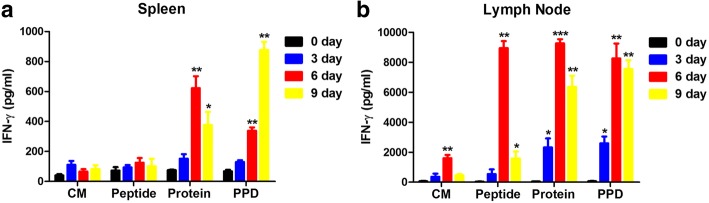
Detection of IFN-γ production following BCG infection. Four groups of C57BL/6 mice (*n* = 6) s.c. vaccinated with 1 × 10^8^ CFU BCG were sacrificed at different time points, and their spleens and inguinal LNs were removed. Increased IFN-γ levels were detected in the culture supernatants of splenocytes (**a**) and inguinal LN cells (**b**). Results are representative of three independent experiments and presented as means ± SEM. Statistical significance was determined using Student’s *t*-test (*P < 0.05, **P < 0.01, ***P < 0.001). CM, culture medium

In order to evaluate the kinetics of the Ag85A-specific T-cell immune response to BCG infection, mononuclear cells isolated from BCG-immunized mice were stimulated in vitro with Ag85A peptide, Ag85A protein, or bovine purified protein (PPD), and concentrations of IFN-γ in culture supernatants were measured. The result showed a significant increase in Ag85A-specific IFN-γ production by inguinal LN mononuclear cells 3 days after BCG injection, with an even greater increase after 6 days (Fig. 1). Ag85A-specific T lymphocytes in both the spleen (Fig. 1a) and inguinal LN (Fig. 1b) produced high levels of IFN-γ when stimulated with Ag85A, although IFN-γ production was 10-fold higher in the LN group. This suggests that the Ag85A-specific T-cell immune response was initiated in the inguinal LN 6 days following BCG infection. Differences in IFN-γ production in the murine spleen and LN may be a consequence of differences in the frequency of T cells among mononuclear cells.

### Dynamics of DC ag-presenting activity in vivo

**Fig. 2:**
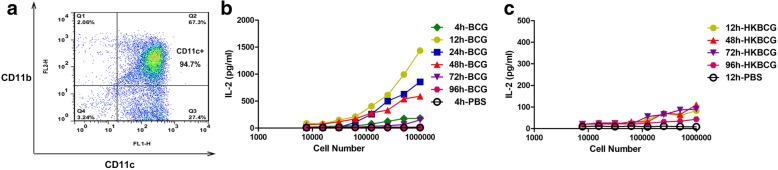
Detection of murine LN DCs Ag-presenting activity ex vivo. Suspensions of inguinal LN cells from C57BL/6 mice were stained with anti-CD11c MicroBeads and separated by autoMACS, resulting in a population of 94.7% CD11c^+^ cells (**a**). To investigate the dynamics of LN DC Ag-presenting activity, we harvested and sorted LN DCs from the inguinal LN at various time points groups after s.c. injection of mice (*n* = 6) with BCG or heat-killed BCG. Then, these cells were serially diluted and used to directly stimulate DE10 T-cell hybridomas. In vivo formation of Ag85A peptide-MHC complexes on LN DCs from mice injected with BCG (**b**) or heat-killed BCG (**c**) were estimated by measuring IL-2 production in DE10 T-cell hybridoma culture supernatants ex vivo. The experiment was repeated at least three times

To investigate the dynamics of inguinal LN DC Ag presentation, we tested their capacity to stimulate DE10 T-cell hybridomas at several time points after subcutaneous injection of mice with BCG. The inguinal LN DCs (CD11c^high^) were sorted by autoMACS with a purity of 94.7% (Fig. 2a). When mice were infected with BCG, LN DCs collected at early time points invoked a response from DE10 hybridomas, with IL-2 being detected following stimulation with those harvested 4 h post-injection, and the highest IL-2 production being observed in response to DCs from mice infected for 12 h. However, IL-2 was only minimally produced in response to DCs from mice infected for 72 h (Fig. 2b). Interestingly, when mice were s.c. injected with heat-killed BCG, Ag-presenting activity markedly decreased from 12 h to 96 h post-injection, suggesting that live BCG is necessary for efficient Ag presentation by DCs in vivo (Fig. 2c). Together, these results indicate that the MHC II presentation of mycobacteria-derived peptides by inguinal LN DCs is only transient, with Ag85A peptide-MHC II complexes on the surfaces of inguinal LN DCs disappearing rapidly.

### Analysis of MHC II, CIITA and T-cell costimulatory molecules on DCs following BCG infection

**Fig. 3:**
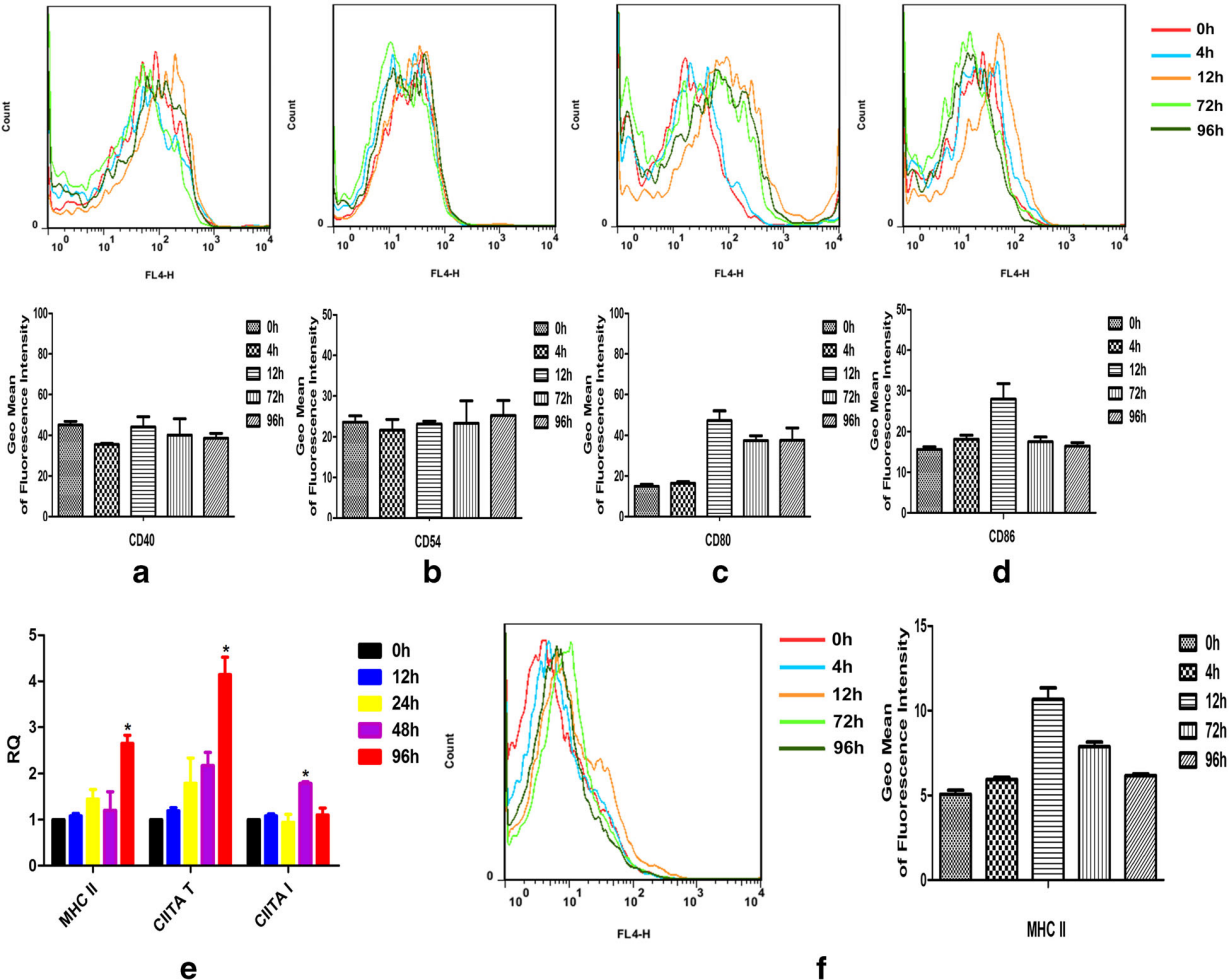
Expression of MHC II, CIITA, and co-stimulatory molecules on DCs following BCG infection. Inguinal LNs were obtained at different time points following s.c. infection of mice with 1 × 10^8^ CFU BCG (*n* = 6) and were sorted and stained with a panel of mAbs to detect cell-surface expression of CD40 (**a**), CD54 (**b**), CD80 (**c**), and CD86 (**d**) by FACS. Inguinal LNs were obtained from five groups of mice (*n* = 6) at different time points following s.c. injection of 1 × 10^8^ CFU BCG. Transcription levels of MHC II, total CIITA (CIITA T), and CIITA type I (CIITA I) were analyzed using real-time PCR (**e**), and DCs were sorted and stained with mAbs to detect MHC II by FACS (**f**). The results are representative of three independent experiments and presented as means ± SEM. Statistical significance was determined using Student’s *t*-test (*P < 0.05)

We measured the expression of cell surface markers involved in Ag presentation and T-cell interaction. Sorted inguinal LN DCs were stained with a panel of monoclonal antibodies (mAbs) to detect CD40, CD54, CD80, and CD86 by Flow Cytometry (FACS). No obvious regulation of CD40 or CD54 on DCs was observed during infection (Fig. 3a, b). High levels of CD80 and CD86 were noted at 12 h, but the presence of these markers had decreased by 72 h and 96 h (Fig. 3c, d). These results indicate that inguinal LN DCs undergo functional activation in the early stages of BCG infection.

We next investigated transcription levels of MHC II and CIITA transcription in inguinal LN DCs during BCG infection using real-time PCR. MHC II expression was found to be increased by BCG infection at a relatively slow rate, while total CIITA transcription was rapidly induced, and expression of CIITA type I declined between 48 and 96 h (Fig. 3e). In addition, to evaluate the expression of MHC II molecules involved in Ag presentation, sorted inguinal LN DCs were stained with mAbs for FACS analysis. All sorted DCs demonstrated up-regulation of MHC II molecules following the initiation of infection (Fig. 3f). The fact that the transcription and expression of MHC II proteins on the cell surface did not decline following BCG infection suggests that the expression and trafficking of MHC class II molecules may be not associated with the rapid loss of Ag85A peptide-MHC II complexes. As a result, it can be deduced that LN DCs do not provide a continuous source of mycobacterial Ag85A peptides for the formation of peptideMHC II complexes.

### BCG infection kinetics of murine LN DCs

**Fig. 4:**
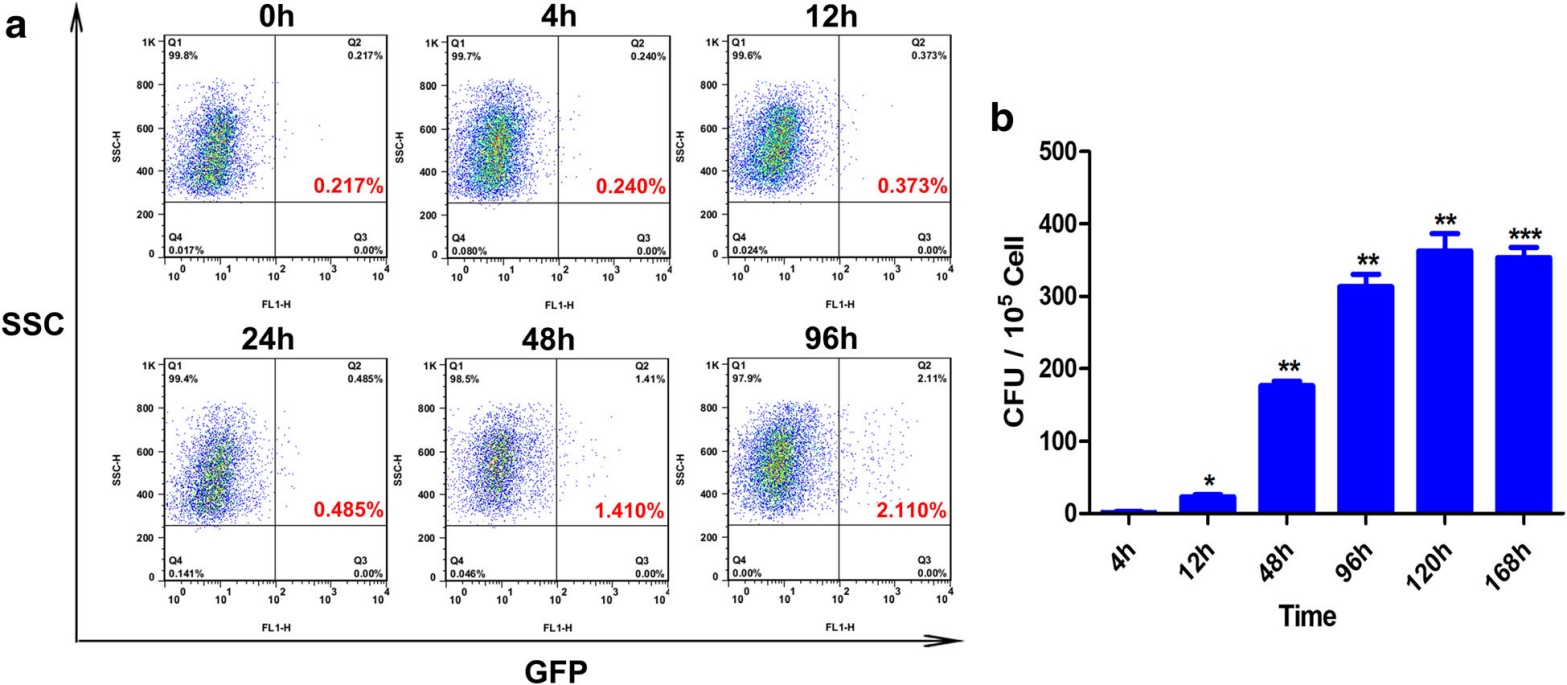
BCG infection kinetics of murine LN DCs. Six groups of mice (*n* = 6) were s.c. injected with 1 × 10^8^ CFU rBCG-GFP, and LN cells were harvested at different time points. DCs were sorted and analyzed for the presence of rBCG-GFP. Infection of murine LN DCs with BCG (**a**). Six groups of mice (*n* = 6) were s.c. injected with 1 × 10^8^ CFU BCG, and BCG in DCs was quantified in CFUs by culturing on Middlebrook 7H10 agar (**b**). The results are representative of three independent experiments and presented as means ± SEM. Statistical significance was determined using Student’s *t*-test (*P < 0.05, **P < 0.01, ***P < 0.001)

In order to monitor the presence of BCG bacilli in inguinal LN DCs following s.c. infection of mice and thus evaluate the infection rate of these immune cells, rBCG-GFP cells were used in combination with FACS. As might be expected, 0.4% of LN DCs exhibited green fluorescence after 12 h of infection, and this figure had increased to 2% by 96 h post-injection (Fig. 4a). We next determined whether BCG survives and multiplies within DCs over the course of infection. CFUs appeared at 4 h and had increased significantly by 12 h after s.c. administration of BCG to mice, and numbers remained elevated until the end of the experiment (Fig. 4b). These results suggest that BCG infected the inguinal LN DC 12 h post-infection and that it survives within the inguinal LN DC pool, representing a continuing source of mycobacterial Ag85A protein, with which LN DCs can form Ag85A peptide-MHCII complexes in vivo.

## Discussion

CD4^+^ T-cell responses and the production of IFN-γ are particularly important to the containment of *Mtb* infection. In mice, between 1 and 3 weeks after initial infection, *Mtb*-specific T cells appear in the lungs, IFN-γ is expressed, and the bacterial burden is controlled [10]. Production of IFN-γ by splenocytes in response to Ag restimulation is observed within 6 days after i.v. *Mtb* infection [11]. To determine when the T-cell response is initiated, we obtained splenocytes and inguinal LN cells from mice 3, 6, and 9 days after s.c. BCG injection. Inguinal LN cells collected 6 days after infection produced IFN-γ in response to Ag85A restimulation. Thus, the T-cell immune response appears to have been initiated in the inguinal LN day 6 following BCG infection.

The mechanisms involved in MHC class II Ag processing and presentation, which are required for CD4^+^ T-cell activation, are crucial for controlling *Mtb* infection. Previous research investigated the kinetics of Ag-presenting activity by harvesting spleens following i.v. administration of rBCG expressing the *E. coli* MalE protein as a reporter Ag. The formation of MalE peptide-MHC complexes in splenic DCs was detected at 2, 4, and 12 h after rBCG infection, while MalE was barely detectable at 48 h [9]. However, this process has not yet been directly examined in LN DCs and by using an endogenous *M. bovis* BCG Ag. To investigate the dynamics of LN DCs Ag presentation, we harvested and sorted these cells from inguinal LNs at several time points after s.c. injection of mice with Ag85A protein or BCG and tested their capacity to stimulate DE10 T-cell hybridomas, which are specific for an immunodominant Ag85A peptide. In this manner, in vivo formation of Ag85A peptide-MHC complexes on DCs from BCG-injected mice was detected by measuring IL-2 production in DE10 T-cell hybridoma culture supernatants ex vivo. Ag85A peptide-MHC complexes on LN DCs appeared rapidly after inoculation, with IL-2 production being detected in response to DCs collected 4 h after BCG infection and the highest production in response to those harvested at 12 h. By contrast, IL-2 levels following exposure to DCs harvested 72 h after infection were barely detectable. Together, these results indicate that the MHC II presentation of mycobacteria-derived peptides by inguinal LN DCs is only transient, with Ag85A peptide-MHC II complexes on the surfaces of inguinal LN DCs disappearing rapidly. Some reports have shown that peptide-MHC complexes have a half-life of 25 h [12]. Thus, it can be concluded that the synthesis of Ag85A peptide-MHC II complexes on inguinal LN DCs was interfered.

Several reports have shown that *Mtb* and *M. bovis* inhibit intracellular processes associated with Ag presentation, including Ag processing, MHC class II expression, the trafficking of MHC class II molecules, and peptide-MHC class II binding [13, 14]. CIITA is the master transcriptional regulator of MHC class II molecules [15]. The transcription of CIITA itself is regulated by the three unique promoters pI, pIII, and pIV, which drive the expression of CIITA types I, III, and IV, respectively. pI is constitutively active in DCs [14]. In the current investigation, LN DCs exhibited the up-regulation of cell-surface MHC II molecules from 4 to 96 h following infection. Using real-time PCR, we analyzed the transcription levels of MHC II, total CIITA, and CIITA type I in DCs in response to BCG. Expression of MHC II was found to be induced by BCG infection relatively slowly, while total CIITA transcription was rapidly induced. This indicates that the transcription and expression of MHC II proteins on the cell surface did not declined following BCG infection, suggesting that the expression and trafficking of MHC class II molecules may be not associated with the rapid loss of Ag85A peptide-MHC II complexes. As a result, it can be deduced that LN DCs do not provide a continuing source of mycobacterial Ag85A peptides for the formation of peptide-MHC II complexes.

Considerable evidence shows that DCs can phagocytose mycobacteria and may be the first cells to encounter such pathogens, therefore, DCs are likely to be responsible for initiating the subsequent immune response. The survival of mycobacteria within DCs has been assessed previously in vitro using the BCG vaccine strain and virulent *M. bovis*, both of which were shown to be phagocytosed by DCs after 24 h of infection [16]. *Mtb* cells disseminate to draining LNs within 8 days following respiratory infection [17]. Approximately 2% of the splenic DC population (CD11c^+^ cells) was found to contain BCG at 4 h following i.v. infection [9]. In the present study, the presence of rBCG-GFP bacilli in inguinal LN DCs following s.c. inoculation of mice was monitored by FACS. As expected, the percentage of infected DCs increased to 2% after 96 h of infection. We then examined whether mycobacteria survive and multiply within DCs during infection. Following s.c. administration of BCG to mice, CFUs appeared at 4 h, increased significantly by 12 h, remaining elevated until the last time point. These results suggest that BCG survives within the inguinal LN DC pool, representing a continuing source of mycobacterial Ag85A protein with which LN DCs can form Ag85A peptide-MHCII complexes in vivo. Some reports have shown that live *Mtb* can alter phagosome maturation and decrease Ag processing, providing a mechanism for *Mtb* to evade immune surveillance and enhance its survival within the host [18–20]. Based on our findings, we conclude that the inhibition of Ag processing due to the reduced production of Ag85A peptide is the primary reason for the rapid loss of Ag85A peptide-MHC II complexes.

## Conclusions

In the present study, we evaluated the in vivo dynamics of early Ag presentation by murine LN DCs in response to *M. bovis* BCG Ag85A protein. Our results showed that the early Ag-presenting activity of murine DCs induced by *M. bovis* BCG Ag85A protein in vivo was transient and that the inhibition of Ag processing induced by a decrease in the production of Ag85A peptide is the primary reason for the rapid loss of Ag85A peptide-MHC II complexes and the short duration of Ag presentation by LN DCs during BCG infection in vivo*.*

## Methods

### Experimental animals

Six-week-old female C57BL/6 mice were purchased from Vital River (Beijing, China). The mice were housed, handled, and immunized at our animal biosafety facilities, and all procedures were approved by the Institutional Animal Experimental Committee of Yangzhou University. All experiments were performed according to the national guidelines for animal welfare. The mice were euthanized by cervical dislocation under isoflurane, and spleens and inguinal LNs were collected for analysis.

### Bacterial strains and culture conditions

*M. bovis* BCG Pasteur 1173P2 and rBCG expressing GFP (rBCG-GFP) were kindly provided by Dr. Xiaoming Zhang (Institut Pasteur of Shanghai, Chinese Academy of Sciences, Shanghai, China). Both strains were grown with gentle agitation (80 rpm) in Middlebrook 7H9 medium (Difco, Detroit, MI, USA) supplemented with 0.05% Tween 80 and 10% albumin-dextrose-catalase (ADC) enrichment or on solid Middlebrook 7H10 medium (Difco) supplemented with 0.05% Tween 80 and 10% oleic-ADC enrichment.

### T-cell hybridoma and Ags

MHC II-restricted DE10 T-cell hybridomas specific for the *Mtb* Ag85A peptide comprising amino acids 241 to 260 [21] were kindly provided by Dr. Claude Leclerc (Institut Pasteur, Paris, France). The Ag85A protein was constructed and expressed in our laboratory, and the Ag85A peptide (amino acids 241–260) was synthesized by SciLight Biotechnology (Beijing, China).

### Detection of IFN-γ production following BCG infection

C57BL/6 mice were s.c. vaccinated with 1 × 10^8^ CFU BCG and sacrificed 3, 6, and 9 days later, at which point, spleens and inguinal LNs were removed aseptically and transferred to complete RPMI-1640 medium for preparation of single-cell suspensions. The mononuclear cells, isolated using Histopaque 1083 (Sigma, St. Louis, MO, USA), were seeded at 1 × 10^6^ cells/well in 96-well plates containing complete RPMI-1640 medium. They were subsequently stimulated with 10 μg/ml Ag85A peptide, 10 μg/ml Ag85A protein, or 5 μg/ml bovine PPD (Prionics, Schlieren, Switzerland) and incubated at 37 °C in an atmosphere of 5% CO_2_ in air. Supernatants were then harvested at 48 h post-stimulation, frozen, and later tested for IFN-γ concentration by sandwich enzyme-linked immunosorbent assay (ELISA, BD Biosciences, Franklin Lakes, NJ, USA).

### Ag presentation assay

C57BL/6 mice were s.c. injected with 1 × 10^8^ CFU BCG or heat-killed BCG in 200 μl PBS or with PBS alone. Mice were sacrificed at various time points, and their inguinal LNs removed and perfused with 400 U/ml collagenase type IV (Invitrogen, Carlsbad, CA, USA) containing 50 μg/ml DNase I (Invitrogen). Single LN-cell suspensions were prepared, and DCs were sorted with an autoMACS separator (Miltenyi Biotec, Bergisch Gladbach, Germany) using CD11c as a cell marker. Specifically, LN cells were first incubated with anti-CD11c MicroBeads (Miltenyi Biotec) before autoMACS separation, resulting in a population of CD11c^high^ cells (DCs). The purity of these murine LN DCs was then analyzed using a FACSCalibur instrument (BD Biosciences). For the ex vivo Ag presentation assay itself, the purified LN DCs were transferred to 96-well microplates and serially diluted in complete RPMI-1640 medium. DE10 T-cell hybridomas at a density of 1 × 10^5^/well were then added, and after incubation for 24 h, supernatants were collected, frozen, and later tested for IL-2 content by sandwich ELISA (BD Biosciences).

### Cell phenotype analysis

C57BL/6 mice were s.c. injected with 1 × 10^8^ CFU BCG in 200 μl PBS or with PBS alone. The mice were sacrificed after various periods for the preparation of single inguinal LN-cell suspensions and sorting of DCs by autoMACS. FITC-conjugated anti-CD11c, and biotinylated anti-I-Ad, anti-CD40, anti-CD54, anti-CD80, and anti-CD86 antibodies were used to label cells. Allophycocyanin-conjugated streptavidin was employed to visualize biotin conjugates. A FACSCalibur and FlowJo software (FlowJo LLC, Ashland, OR, USA) were then used for multicolor staining analysis of the labeled cells. DCs were sorted using the autoMACS system before being pelleted and resuspended in lysis buffer. Cellular RNA was purified with an RNeasy kit (Qiagen, Valencia, CA, USA) according to the manufacturer’s instructions, and total RNA was reverse-transcribed into cDNA using SuperScript reverse transcriptase (Thermo Fisher Scientific, Waltham, MA, USA).

### In vivo infection assay

C57BL/6 mice were s.c. injected with 1 × 10^8^ CFU BCG or rBCG-GFP in 200 μl PBS or with PBS alone. Mice were sacrificed at various time points, and single inguinal LNs were removed aseptically and transferred to complete RPMI-1640 medium. Single-cell suspensions were prepared, and DCs were sorted with an autoMACS separator as above. The percentage of DCs infected with rBCG-GFP was analyzed using a FACSCalibur instrument and FlowJo software. BCG-infected DCs were pelleted and resuspended in lysis buffer. Ten-fold serial dilutions of these suspensions were then plated on solid Middlebrook 7H10 medium, and colonies were counted after incubation at 37 °C for 2–3 weeks.

### Statistical analysis

All data are expressed as means ± SE. Statistical analysis was performed by Student’s *t*-test using GraphPad Prism software. *P* values < 0.05 were considered statistically significant.

## Abbreviations

ADC: Albumin-dextrose-catalase
APC: Antigen-presenting cell
BCG: Bacillus Calmette–Guérin
CIITA: MHC class II transactivator
DC: Dendritic cell
LN: Lymph node
MHC II: Major histocompatibility complex class II
*Mtb*: *Mycobacterium tuberculosis*
PPD: Purified protein derivative
rBCG: Recombinant BCG
TB: Tuberculosis
